# Black Salve: A Dangerous Corrosive Disguised as an Alternative Medicine

**DOI:** 10.7759/cureus.41248

**Published:** 2023-07-01

**Authors:** Andrew J Ordille, Ashley Porter, Amy M Scholl

**Affiliations:** 1 Biomedical Sciences, Cooper Medical School of Rowan University, Camden, USA; 2 Internal Medicine, Cooper University Hospital, Camden, USA

**Keywords:** skin infections, sanguinaria, bloodroot, secondary infection, complementary & alternative medicine, black salve

## Abstract

Black salve is a dangerous compound that has long been used as an alternative and complementary medicine despite clear warnings of its hazards from the medical community and governmental agencies. A paucity of information exists for clinicians seeking guidance regarding the management of black salve-related adverse outcomes. Secondary infection is a common sequela of black salve application to skin lesions. This case report presents a summary of the management of a secondary infection in a patient who applied black salve to an open skin wound. The resolution of this patient’s symptoms was a function of the interdisciplinary care provided by infectious disease specialists, an acute surgical care team, and dermatologists. The patience, clinical expertise, and judgment provided by these healthcare teams resulted in an appropriate diagnosis while also avoiding unnecessary medical procedures. This case sheds light on one of the varied consequences of black salve use and advocates for the incorporation of multiple medical teams in the management of black salve-related events.

## Introduction

Black salve is an unregulated topical corrosive that has been used as an alternative medicine for treating a range of dermatologic issues, including moles, polyps, warts, scars, burns, and malignancies. While there are a variety of formulations that incorporate various herbs and flowering plants, its composition is primarily derived from two core ingredients: bloodroot (*Sanguinaria canadensis*) and the inorganic chemical compound zinc chloride (ZnCl_2_) [1]. This herbal paste’s deleterious mechanism of action is a function of active metabolites derived from sanguinarine, a benzylisoquinoline alkaloid within the bloodroot, and escharotic properties extracted from the synthetic corrosive compound zinc chloride (ZnCl_2_) [2]. The combination of these compounds enacts a topical dermatologic reaction that causes tissue destruction and the formation of an eschar, which is later sloughed away. Histological analyses of black salve-affected areas confirm its escharotic properties with prominent dermal scaring, extensive ulceration, and tissue necrosis observed microscopically [3].

The history of black salve being viewed as a complementary and alternative medicine can be traced back to the mid-1800s when American surgeon Jesse Weldon Fell suggested the herbal remedy could be used to treat skin cancer [4]. In the years that followed, entrepreneurs and business opportunists would attempt to market, sell, and disseminate information regarding this misbranded and unapproved herbal compound. By 1950, the Food and Drug Administration (FDA) would block the shipment and distribution of all black salve formulations due to the corrosive properties of black salve formulations and consumer risks of delayed healthcare treatment [2, 5]. Despite regulatory efforts and FDA-sponsored campaigns to spread information on its dangers, black salve continues to be used as a home remedy treatment for a variety of skin conditions, including boils, moles, and skin tags. Reports even cite individuals attempting to cure skin cancer with the application of bloodroot products, although both the FDA and American Academy of Dermatology state there is no credible evidence to support this [5-7]. Often, dissemination of evidence-lacking information is driven via online channels, which permits harmful outcomes in individuals unaware of the dangerous effects of black salve. Commonly reported adverse outcomes observed with black salve use include burning pain, abnormal skin pigmentation, ulceration, scarring, and secondary infection [8].

While there have been a handful of reports warning clinicians of the harmful effects of this corrosive commercial product, there exists hardly any information on the management of its adverse effects. The sequelae of changes seen with black salve use are related to the formation of an eschar along with accompanying tissue necrosis and infection [9]. Appropriate management and evaluation of these negative outcomes, such as secondary infection, is necessary to prevent morbidity and mortality. To date, there exists no antidote, reversal agent, or guidelines in the management of black salve-related complications. Many of the clinical decisions made regarding management are made based on symptom reduction and clinical expertise. Timely management of medical conditions, including the dermatologic issues seen with black salve application, must balance risk and reward to avoid unnecessary procedures and treatments. Furthermore, management should incorporate early involvement of multiple care teams, as early involvement of medical sub-specialists has been shown to minimize unnecessary investigations [10]. Here, we present the case of a patient who developed a secondary infection as an unintended consequence of black salve application. The purpose of this article was to reinforce the dangers of black salve use and also to demonstrate the benefits of exercising patience and an interdisciplinary team approach in managing atypical dermatologic issues.

## Case presentation

A 46-year-old woman with no past medical history presented to an outside hospital with erythema and tenderness of her right axilla. She was a current tobacco smoker with a 10 pack-year history and denies any history of intravenous drug use. She had no known drug or environmental allergies and took no medications or supplements. In the two weeks prior to her presentation, the patient had a tender, pustular, raised lesion in her right axilla, which she attempted to drain by shaving the area and self-manipulating with pressure. Following eruption of it, the patient applied black salve to the open wound. The right axilla quickly became erythematous, and the patient reported having accompanying fevers, chills, and night sweats which prompted an evaluation at the emergency department.

On initial examination, the patient had a temperature of 37.6^o^C (99.7^o^F) with other vital signs within normal limits. On physical examination, the patient was oriented without focal neurologic or musculoskeletal deficits. Initial laboratory analysis showed a white blood cell (WBC) count of 18.0 x 10^3^/μL (segmented neutrophils, 71.9%; band neutrophils, 7.0%; lymphocytes, 10.5%), a hemoglobin concentration of 12.3 g/mL, a platelet count of 234 x 10^3^/μL, and a lactate level of 3.1 mmol/L. Hepatic and renal function tests were within normal limits with an alanine aminotransferase (ALT) level of 20 U/L, aspartate aminotransferase (AST) of 31 U/L, creatinine of 0.75 mg/dL, and a blood urea nitrogen (BUN) of 7 mg/dL. Blood cultures were subsequently collected. Work-up for an immunocompromised state with screenings for human immunodeficiency virus (HIV), rapid plasma reagin (RPR), and hepatitis yielded negative results. Due to suspicion of skin and soft tissue infection (SSTI), the patient was started on vancomycin and clindamycin.

**Figure 1:**
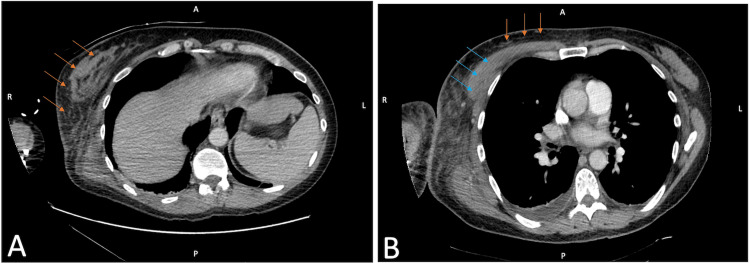
Serial axial plane CT scans with contrast of the patient’s chest. On hospital day one (A), there is an extensive area of subcutaneous edema in the right axilla along the right chest wall (orange arrows) concerning for cellulitis. On hospital day three (B), there is interval worsening of subcutaneous soft tissue findings with extension to the central anterior chest wall (orange arrows) and a new infiltrate (blue arrows) involving the chest wall musculature with interstitial edema, indicating a possible pyomyositis. A = anterior; P = posterior; R = right; L = left

**Figure 2:**
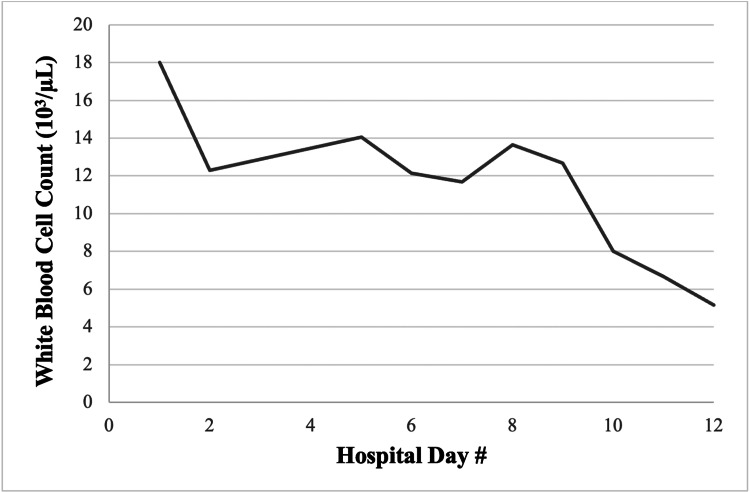
White blood cell monitoring throughout the patient's hospital course.

Initial imaging on admission with computed tomography (CT) showed extensive subcutaneous edema within the right axilla concerning for cellulitis (Figure 1A). Repeat imaging two days later demonstrated new interstitial edema with extension to the chest wall now concerning for pyomyositis (Figure 1B). The area of erythema and soft tissue had expanded since admission, but the patient continued to remain hemodynamically stable with a stable leukocytosis (Figure 2). There was no microbial growth in the collected blood cultures. Levofloxacin was subsequently added for additional antimicrobial coverage. On her fifth day of hospitalization, the patient was transferred to our hospital for a higher level of care due to the progression of physical exam and radiographic findings.

**Figure 3:**
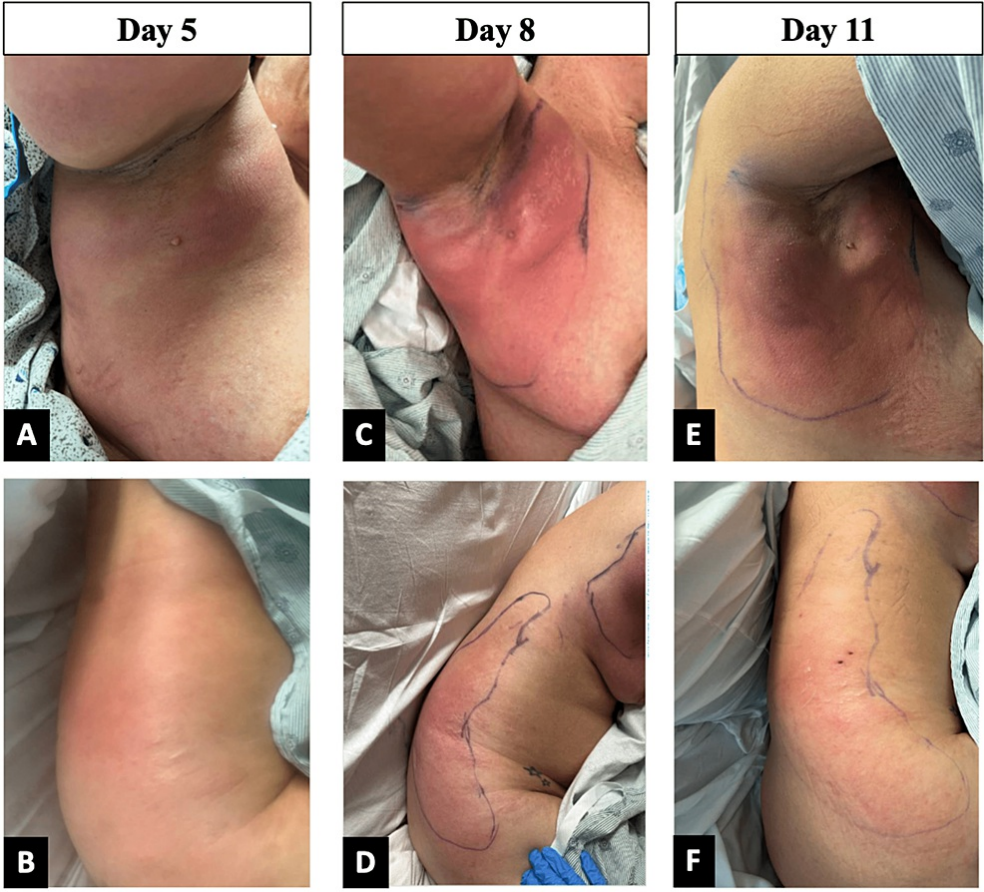
Progression of the patient’s dermatologic findings at the time of transfer (Images A and B), day 8 of hospitalization (Images C and D), and day 10 of hospitalization (Images E and F). Images were taken under the patient's right axilla (Images A, C, and E) and the right flank (Images B, D, and F).

**Figure 4:**
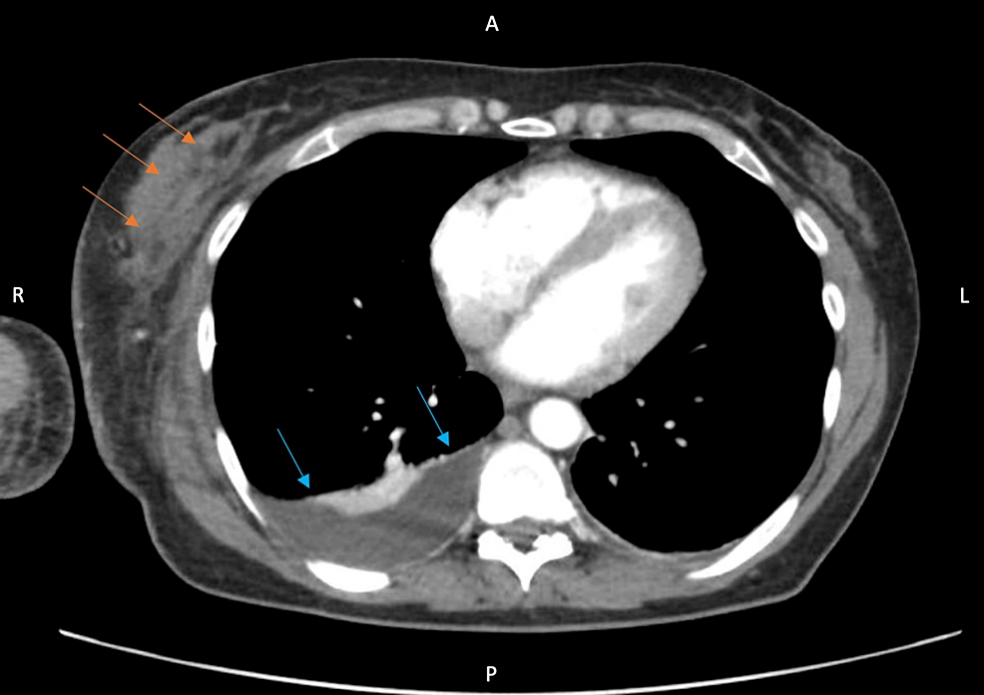
Axial plane CT scan with contrast of the patient’s chest on hospital day eight which demonstrates a new low attenuating focus in the right pectoralis (orange arrows), which could reflect fat necrosis or abscess formation. Also observed is a small- to moderate-sized right pleural effusion (blue arrows). A = anterior; P = posterior; R = right; L = left

At the time of transfer, initial evaluations were made by healthcare providers from infectious disease, acute care surgery, and dermatology services (Figure 3A, 3B). Due to lack of evidence for muscle involvement, the patient was presumed to have cellulitis. A level of suspicion was maintained for other etiologies, including pyomyositis, myonecrosis, and inflammatory carcinoma. Creatine kinase levels trended over the patient’s hospital course and remained within normal limits. After transfer, there was increased erythema, edema, tenderness, and induration which had discontinuously extended to the right flank and right hip (Figure 3C, 3D). CT imaging on day eight of hospitalization demonstrated fat infiltration and possible abscess formation (Figure 4). A punch biopsy of the affected right hip was collected, which revealed inflammatory changes consistent with a soft tissue infection. No bacteria, fungi, or acid-fast organisms were cultured. Dermatology indicated that a deeper resection of the indurated areas within the right axilla via wedge biopsy might be necessary, yet acute surgical care continued to be withheld.

On day nine, antibiotic coverage was changed to now include cefepime, metronidazole, and linezolid. The following day, there was a reduction in the erythema and tenderness of the right axilla, flank, and hip. Blood cultures collected on admission from outside hospital as well as those collected upon transfer continued to demonstrate absent microbial growth. Cultures of the affected skin areas for either methicillin-sensitive or methicillin-resistant *Staphylococcus aureus* were negative. On day 12 of hospitalization, there was a reduction in edema and significant improvement in the patient’s dermatologic findings (Figure 3E, 3F). Ultrasounds confirmed there was no drainable fluid collection or abscess, and the patient was discharged with instruction to take amoxicillin/clavulanate for seven days.

## Discussion

Despite various regulatory efforts, medical cautions, and government-sponsored information campaigns to halt lay use of this dangerous and corrosive compound as an alternative and commentary medicine, the widespread use of black salve persists. Given this, physicians need to be aware of the sequela of changes that occur with black salve use to provide timely and appropriate care to inflicted patients. This case report provides an example of the management of a particular side effect reported with black salve use secondary infection [8]. The patient presented here had a rapidly progressive skin infection, which raised concerns for an immunocompromised state. The evolution in the appearance (i.e., erythema, induration), area of involvement, and associated pain raised further suspicion for extension of skin infection to soft-tissue compartments. Although a final diagnosis of cellulitis would be established, a high level of suspicion for pyomyositis was maintained throughout the patient’s hospital course.

To date, there have been at least 36 patients who have attempted to treat skin lesions with black salve, with many having experienced an adverse cosmetic outcome [1]. Cienki et al. described a case of an enterocutaneous fistula in a man with metastatic colon cancer following black salve application to the abdominal wall subcutaneous nodule [11]. There are multiple reports of black salve application to a localized cancerous lesion with subsequent progression of the primary lesion to a metastatic process [12, 13]. Ulceration is a commonly reported finding observed in patients who apply this escharotic substance to skin lesions as an herbal cure [11, 14]. The patient’s diagnosis is congruent with previous reports of black salve-related skin and soft tissue infections, as black salve application has been shown to cause cellulitis in the adjacent areas of the application [9]. These reports highlight the wide-ranging and medically significant outcomes associated with black salve application.

The resolution of this patient’s symptoms was a function of interdisciplinary team care. The early involvement of infectious disease specialists, surgical care, and dermatologists resulted in appropriate antimicrobial coverage, successive radiological evaluations, and histologic analysis of tissue samples, respectively. Each action contributed to the appropriate diagnosis and treatment of the patient’s condition while also avoiding unnecessary medical procedures and healthcare expenses. The patient’s presenting leukocytosis was countered with an antibiotic regimen that reduced the white blood cell count. Despite the rapid correction of leukocytosis, infectious disease consultants remained actively involved in the patient's care, providing multiple antibiotic changes and daily evaluations of the patient. The results of the radiographic testing from the outside hospital prompted the need for elevation of care due to concerns of soft tissue infection (i.e., pyomyositis). Multiple evaluations by the surgical service team would refute this SSTI concern and subsequently prevent the patient from obtaining unnecessary procedures, including a wedge biopsy or debridement. Lastly, the ability of the patient to receive daily evaluations by dermatologists after transfer allowed for the patient to receive an appropriate diagnosis of cellulitis, which was made with evidence provided from the punch biopsy, radiographic imaging, and physical examination findings and guided by clinical expertise.

Although this patient’s clinical course was a result of infectious insult, there is a possibility that the changes seen were due solely to black salve corrosive damage. Importantly, the patient’s improved clinical course, despite not having a surgical wedge biopsy or debridement, highlighted the importance of exercising patience in potential black salve-related secondary skin infections. This case sheds light on secondary infection, one of the varied consequences of black salve use, with appropriate management guided by interdisciplinary team care and patience in medical decision-making.

## Conclusions

Black salve is a topical corrosive disguised as an alternative and complementary medicine. Despite multiple reports of its dangers and regulatory efforts made by governmental agencies, black salve continues to be used as a home remedy for a variety of dermatologic findings. One of the common side effects associated with black salve use is secondary infection, including cellulitis. Despite the paucity of guidance available on managing black salve-related skin and soft tissue infections, clinicians must maintain a high level of suspicion in treating black salve complications. Management of atypical skin lesions, like those seen with black salve, should involve multiple disciplines within health care to provide appropriate and effective treatments guided by clinical expertise.
